# International External Quality Assessment of Molecular Detection of Rift Valley Fever Virus

**DOI:** 10.1371/journal.pntd.0002244

**Published:** 2013-05-23

**Authors:** Camille Escadafal, Janusz T. Paweska, Antoinette Grobbelaar, Chantel le Roux, Michèle Bouloy, Pranav Patel, Anette Teichmann, Oliver Donoso-Mantke, Matthias Niedrig

**Affiliations:** 1 Robert Koch Institute, Centre for Biosafety, Berlin, Germany; 2 Center for Emerging and Zoonotic Diseases, National Institute for Communicable Diseases of the National Health Laboratory Service, Sandringham, South Africa; 3 Division Virology and Communicable Disease Surveillance, School of Pathology, University of the Witwatersrand, Johannesburg, South Africa; 4 Pasteur Institute, Paris, France; University of North Carolina at Chapel Hill, United States of America

## Abstract

Rift Valley fever (RVF) is a viral zoonosis that primarily affects animals resulting in considerable economic losses due to death and abortions among infected livestock. RVF also affects humans with clinical symptoms ranging from an influenza-like illness to a hemorrhagic fever. Over the past years, RVF virus (RVFV) has caused severe outbreaks in livestock and humans throughout Africa and regions of the world previously regarded as free of the virus. This situation prompts the need to evaluate the diagnostic capacity and performance of laboratories worldwide. Diagnostic methods for RVFV detection include virus isolation, antigen and antibody detection methods, and nucleic acid amplification techniques. Molecular methods such as reverse-transcriptase polymerase chain reaction and other newly developed techniques allow for a rapid and accurate detection of RVFV. This study aims to assess the efficiency and accurateness of RVFV molecular diagnostic methods used by expert laboratories worldwide. Thirty expert laboratories from 16 countries received a panel of 14 samples which included RVFV preparations representing several genetic lineages, a specificity control and negative controls. In this study we present the results of the first international external quality assessment (EQA) for the molecular diagnosis of RVF. Optimal results were reported by 64% of the analyses, 21% of the analyses achieved acceptable results and 15% of the results revealed that there is need for improvement. Evenly good performances were achieved by specific protocols which can therefore be recommended as an accurate molecular protocol for the diagnosis of RVF. Other protocols showed uneven performances revealing the need for improved optimization and standardization of these protocols.

## Author Summary

Rift Valley fever (RVF) is a zoonotic viral disease posing an increasing threat to animals and humans worldwide. Recent severe outbreaks of the disease in animal and human populations in endemic regions and outside the disease's traditional geographic boundaries necessitate the need for evaluating the diagnostic performance of RVF expert laboratories. Molecular methods are increasingly used for a rapid and accurate detection of viral nucleic acid. In this study we present the results of the first international external quality assessment (EQA) for the molecular diagnosis of RVF. Such EQA studies allow participating laboratories to monitor the quality and identify possible weaknesses of current diagnostic methods. Participants to this RVF EQA were 30 expert laboratories from 16 different countries worldwide. The study demonstrated that optimal results could be achieved by the majority of laboratories. Specific protocols showed evenly good performances and can therefore be recommended to all expert laboratories. However, other methods showed uneven performances suggesting the need for improved optimization and standardization of these protocols.

## Introduction

Rift Valley fever (RVF) is a mosquito-borne viral zoonosis that primarily affects animals but also has the capacity to infect humans. An epizootic of RVF is usually first indicated by a wave of unexplained abortions as infected pregnant livestock abort virtually 100% of fetuses. The disease is less fatal to humans as most human infections are asymptomatic and when clinical symptoms appear they are in majority influenza-like. Nevertheless, some cases may develop a severe RVF disease with variable clinical signs. More severe cases occur in 2% of the RVF cases and fall into three categories: liver necrosis with hemorrhaging, retinitis with visual impairment and meningoencephalitis [1], [2].

The causative agent of RVF, the RVF virus (RVFV), is a negative-stranded RNA virus, a member of the genus *Phlebovirus* of the *Bunyaviridae* family. The number of identified viral lineages of RVFV has increased from 3 in an early analysis [3] to 7 in a 2007 study [4], and in the most recent report 15 distinct genetic groups were reported [5]. Phylogenetic analysis shows that the virus emerged in the mid-19^th^ century, but it was first identified in 1930 during an outbreak of abortions and deaths among sheep in the Rift Valley region of Kenya. In 1977–78, several millions of people were infected and more than 600 died during a severe epidemic in Egypt [6]. Since then, the geographical distribution of the virus has widely spread and now includes most countries of the African continent as well as Madagascar and the Arabian Peninsula. During the past five years, outbreaks have been reported in Kenya [7], Somalia, Tanzania [8], Sudan [9], Mayotte [10], Madagascar [11], Swaziland, South Africa and Mauritania [12], [13] Another important concern is the increasing number of human fatalities during the most recent outbreaks [14].

The emergence or re-emergence of RVFV activity is periodic and associated with exceptionally heavy rainfalls which allow massive breeding of flood-water *Aedes* mosquitoes with the capacity for transovarial transmission [15] and other competent vectors such as *Anopheles* and *Culex* species [9]. These mosquitoes may initiate outbreaks among livestock, particularly breeds of cattle and sheep. The virus can be transmitted to humans by mosquito bite or by contact with infected tissues of domestic and wildlife ruminants. The sudden onset of large numbers of abortions and fatalities in RVFV affected livestock, resulting in the virus spread to humans can greatly strain public health and veterinary infrastructures.

Unavailability of effective antiviral drugs and commercial vaccines for human or animal use outside endemic countries, including the US and Europe, and the recent spread of RVFV beyond its usual boundaries has resulted in increased international demand for qualified diagnostic tools for a rapid and accurate diagnosis of RVF.

Diagnostic methods for RVFV detection include virus isolation [16], antigen [17], [18] and antibody detection methods [19]–[21] and nucleic acid amplification techniques. Isolation procedures are expensive, time-consuming and require high biocontainment facilities. Serological methods such as antigen or antibody-detection enzyme immunoassays (EIA) require several samples and often lack sensitivity. Therefore, considerable efforts have been made to develop molecular methods which allow a rapid, accessible and accurate detection of RVFV. The use of direct diagnostic methods such as molecular methods, can detect the disease during the acute phase of the infection thus allowing efficient patient management, avoiding nosocomial cases and providing rapid outbreak response. Highly sensitive nucleic acid detection methods have been developed including polymerase chain reaction (PCR) assays such as reverse-transcriptase PCR (RT-PCR) [22], real-time RT-PCR (qRT-PCR) [23]–[25] and more recently real-time reverse-transcription loop-mediated isothermal amplification (RT-LAMP) [26] and recombinase polymerase amplification assays (RPA) [27].

The performance of the different techniques applied for molecular diagnosis of RVFV may vary between laboratories. External quality assessment (EQA) studies to assess the quality of RVFV molecular diagnostics have not been performed until now. The EQA study allows the participating laboratories to monitor the quality of current diagnosis, identify possible weaknesses of particular diagnostic methods and evaluate their capacity for surveillance activities. Therefore the first EQA study for the molecular diagnosis of RVFV was organized by the European Network for Diagnostics of ‘Imported’ Viral Diseases (ENIVD) (http://www.enivd.org) in 2012. Using the results of this study, the ENIVD can also provide support and advice to all laboratories performing RVFV molecular diagnosis.

## Materials and Methods

### Call for participation

A total of 33 laboratories involved in diagnostics of RVF infections were invited to participate in this study. Invitees were selected from the register of ENIVD members, national/regional reference laboratories for RVF or vector-borne diseases as well as on the basis of their contributions to the literature relevant to this topic. The participation to the study was open and free of charge and included publication of the results in a comparative and anonymous manner. This EQA was coordinated by the ENIVD following comparable procedures used during previous studies performed by the network [28], [29].

### Specimen preparation

A proficiency test panel of 14 samples was prepared which included inactivated and stable RVFV preparations generated from Vero E6 cell culture supernatants of different RVFV genetic lineages and origin. Viral cell supernatants were inactivated by heating for 1 h at 60°C and gamma irradiation (25 kilogray) to assure their non-infectivity. A serum sample spiked with Toscana virus, another phlebovirus, was included as a specificity control as well as two negative controls. The RVFV positive samples selected for this EQA panel are detailed in Table 1. Two dilutions of sample Tambul/Egypt/1994 and 5 dilutions of sample F057/Kenya/2007 were obtained by serial 10-fold dilutions and included in the panel for sensitivity testing.

**Table 1:** Proficiency panel sample composition.

Sample name	Isolate	Lineage	Year	Country	Origin	Accession n°
F057 Kenya 2007	SPU22/07/057	C	2007	Kenya	human	-
Tambul Egypt 1994	94EG Tambul	A	1994	Egypt	ovine	HM587042
South Africa 1981, 20368	Ar20364	F	1981	South Africa	mosquito	HM587101
825/79 Zimbabwe 1979	VRL825/79	C	1979	Zimbabwe	bovine	HM587071
CAR R 1662, CA. Rep. 1985	CAR R1662	G	1985	Central African Republic	human	HM587086
AR 21229, Saudi Arabia 2000	Ar 21229	C	2000	Saudi Arabia	mosquito	-

Genetic lineages referred to as described by Grobbelaar *et al* [5].

All virus material used for the preparation of the EQA panel was obtained from cell culture and not from clinical samples of infected patients. Therefore, there is no requirement for any ethical statement in this study.

All samples were diluted with fresh thawed human plasma previously confirmed as negative for RVFV. Aliquots of 100 µl were number-coded, freeze dried for 24 h (Christ, AlphaI-5, Hanau, Germany) and stored at 4°C until dispatched.

### Validation and dispatch of the panel sets

Before dispatching the panels, 3 different sets of EQA samples were tested and validated by 2 expert laboratories. For validation, the samples were resuspended in 100 µl of water and the RNA extracted using the QIAamp viral RNA minikit (Qiagen, Hilden, Germany). The number of RVFV genome copies present in these samples was determined by qRT-PCR.

Panel samples were shipped by regular post at ambient temperature. We requested participant laboratories to resuspend the samples in 100 µl of water and to analyze the material as serum samples for nucleic acid detection of RVFV following their routine protocols. The EQA panels were distributed to participants with documentation including full instructions and an evaluation form to fill in their results. Participants were also asked to report information on the adopted protocol, the type of RVFV strain and the number of genome copies in each sample when possible as well as any problems encountered concerning the shipment or the packaging of the samples.

### Evaluation of the results

To guarantee anonymous participation, an individual numerical identification code was assigned to the results reported by each laboratory. This number was followed by a letter (a, b, c) when distinguishable data sets of results based on different methods were sent.

The results were scored in reflection of analytical sensitivity and specificity as in previous EQA studies performed by the ENIVD [29], [30]. We assigned one point for correct positive or negative result whereas false-negative/-positive results were not scored. Equivocal or borderline results were not counted as molecular diagnostic methods should always provide a clear positive or negative result.

Results were classified as:

- Optimal when all results were correct
- Acceptable when all correct results are reported except one false-negative result
- Need for improvement when one or more false-positives and/or several false-negative results were reported.

## Results

We obtained from the invitees a response rate of 91% representing a total of 30 participating laboratories from 16 different countries (10 European, 2 African, 3 Middle-Eastern/Asian countries and one American country):

CODA-CERVA, Department of Virology, Epizootic Diseases Section, Uccle, Belgium; ANSES, Virology Unit, Laboratory of Lyon, France; CIRAD, Department BIOS «Control of exotic and emerging diseases», Montpellier, France; IRBA-IMTSSA, Virology Unit, Le Pharo, Marseille, France; BNI, National Reference Centre for Tropical Infectious Diseases, Hamburg, Germany; Bundeswehr Institute of Microbiology, Munich, Germany; Institute for Novel and Emerging Infectious Diseases Friedrich-Loeffler-Institut, Germany; Robert Koch Institute, Berlin, Germany; Institute of Virology, Georg-August University, Gottingen, Germany; Central Virology Laboratory, Ministry of Health, Public Health Laboratories Sheba Medical Center, Israel; Army Medical and Veterinary Research Center, Rome, Italy; Department of Infectious, Parasitic and Immune-Mediated Diseases, Istituto Superiore di Sanità, Rome, Italy; Padiglione Baglivi National Institute for Infectious Diseases “L. Spallanzani”, Rome, Italy; Department of Histology, Microbiology and Medical Biotechnologies, University of Padova, Italy; Center for Vectors and Infectious Diseases Research, National Institute of Health, Aguas de Moura, Portugal; King Fahd Medical Research Center, King Abdulaziz University, Saudi Arabia; Arboviruses and viral hemorrhagic fever Unit, Institut Pasteur de Dakar, Senegal; Defense Medical & Environmental Research Institute, DSO National Laboratories, Singapore; Institute of Microbiology and Immunology, Faculty of Medicine, University of Ljubljana, Slovenia; Onderstepoort Veterinary Institute, South Africa; Deltamune (Pty) Ltd, Centurion, Gauteng, South Africa; Special Viral Pathogens Laboratory, National Institute for Communicable Diseases, South Africa; Laboratory of Arboviruses and Imported Viral Diseases, National Center for Microbiology, Instituto de Salud Carlos III, Spain; National Institute for Agricultural Research and Experimentation (INIA), Madrid, Spain; Viral Diseases Unit, CReSA, Barcelona, Spain; Swedish Institute for Infectious Disease Control, Sweden; Virology group, Spiez Laboratory, Switzerland; Laboratory of Virology, University Hospitals of Geneva, Switzerland; WHO Collaborative Centre for Virus Reference and Research (Arboviruses & VHFs), Health Protection Agency, United Kingdom; Viral Special Pathogens Branch, Infectious Diseases, CDC, Atlanta, United States of America.

A total of 39 datasets were received including 5 double sets from laboratories using 2 methods (lab #6, 7, 21, 27 and 28) and 2 triple sets from lab #5 and #14. Methods used by the same laboratory could differ from the type of technique, the protocol used for a specific technique or the type of instrument used for a specific protocol.

Performances varied among laboratories and scores ranged from 7 to the maximum value of 14. Optimal results were reported by 64% (n = 25) of the analyses; 21% (n = 8) of the analyses achieved acceptable results due to the inability to detect one positive sample, and 15% (n = 6) revealed several false negative and/or one or more false positive results indicating that there is still need for improvement (Table 2 and 3).

**Table 2:** Results of the EQA for molecular detection of RVFV – Part 1.

origin	F 057 Kenya 2007	F 057 Kenya 2007	F 057 Kenya 2007	F 057 Kenya 2007	F 057 Kenya 2007	Tambul Egypt 1994	Tambul Egypt 1994	20368 South Africa 1981	825/79 Zimbabwe 1979	CAR R 1662 CA. Rep. 1985	AR 21229 Saudi Arabia 2000	Sandfly Fever	neg.	neg.			
lineage	C	C	C	C	C	A	A	F	C	G	C	−	−	−			
[gen cop/ml]	4,8E+06	1,7E+06	3,4E+05	1,9E+05	8,8E+04	3,2E+05	4,3E+04	1,7E+06	2,9E+06	7,2E+05	3,3E+05	−	−	−			
lab n°	#2	#9	#12	#4	#14	#5	#13	#1	#6	#15	#3	#11	#7	#8	score	classification	method
**#1**	+	+	+	+	+	+	+	+	+	+	+	−	−	−	14	Optimal	qRT (23)
**#2**	**+**	**+**	**+**	**+**	**+**	**+**	**+**	**+**	**+**	**+**	**+**	−	−	−	14	Optimal	qRT (22)
**#3**	**+**	**+**	**+**	**+**	**+**	**+**	**+**	**+**	**+**	**+**	**+**	−	−	−	14	Optimal	qRT (23)
**#4**	**+**	**+**	**+**	**+**	**+**	**+**	**+**	**+**	**+**	**+**	**+**	−	−	−	14	Optimal	qRT (23)
**#5b**	**+** *	**+** *	**+** *	**+** *	**+** *	**+** *	**+** *	**+** *	**+** *	**+** *	**+** *	−	−	−	14	Optimal	qRT (22) SC
**#5c**	**+**	**+**	**+**	**+**	**+**	**+**	**+**	**+**	**+**	**+**	**+**	−	−	−	14	Optimal	qRT (22) LC
**#6a**	**+**	**+**	**+**	**+**	**+**	**+**	**+**	**+**	**+**	**+**	**+**	−	−	−	14	Optimal	qRT (34) ABI
**#6b**	**+**	**+**	**+**	**+**	**+**	**+**	**+**	**+**	**+**	**+**	**+**	−	−	−	14	Optimal	qRT (ih)
**#7a**	**+**	**+**	**+**	**+**	**+**	**+**	**+**	**+**	**+**	**+**	**+**	−	−	−	14	Optimal	qRT (22) LC
**#8**	**+**	**+**	**+**	**+**	**+**	**+**	**+**	**+**	**+**	**+**	**+**	−	−	−	14	Optimal	qRT (22)
**#10**	**+**	**+**	**+**	**+**	**+**	**+**	**+**	**+**	**+**	**+**	**+**	−	−	−	14	Optimal	qRT (ih)
**#11**	**+**	**+**	**+**	**+**	**+**	**+**	**+**	**+**	**+**	**+**	**+**	−	−	−	14	Optimal	qRT (23)
**#12**	**+**	**+**	**+**	**+**	**+**	**+**	**+**	**+**	**+**	**+**	**+**	−	−	−	14	Optimal	qRT (23)
**#13**	**+**	**+**	**+**	**+**	**+**	**+**	**+**	**+**	**+**	**+**	**+**	−	−	−	14	Optimal	qRT (22)
**#14a**	+	+	+	+	+	+	+	+	+	+	+	−	−	−	14	Optimal	qRT (34) ABI
**#16**	+	+	+	+	+	+	+	+	+	+	+	TOSV+	−	−	14	Optimal	qRT (22)
**#19**	**+**	**+**	**+**	**+**	**+**	**+**	**+**	**+**	**+**	**+**	**+**	−	−	−	14	Optimal	qRT (23)
**#20**	**+**	**+**	**+**	**+**	**+**	**+**	**+**	**+**	**+**	**+**	**+**	−	−	−	14	Optimal	qRT (22)
**#21a**	**+**	**+**	**+**	**+**	**+**	**+**	**+**	**+**	**+**	**+**	**+**	−	−	−	14	Optimal	qRT (22)
**#24**	+	+	+	+	+	+	+	+	+	+	+	−	−	−	14	Optimal	qRT (ih)

+: positive.

−: negative.

bold: quantified result.

* : correct strain.

TOSV: Toscana virus.

RT: reverse transcription.

qRT: real-time RT-PCR.

(24): Drosten et al., 2002.

(25): Bird et al., 2008.

(31): Weidmann et al. 2008.

SC: SmartCycler from Cepheid.

LC: LightCycler from Roche Applied Science.

ABI: 7500 Real-Time PCR System from Applied Biosystems.

(ih): in house assay.

**Table 3:** Results of the EQA for molecular detection of RVFV – Part 2.

origin	F 057 Kenya 2007	F 057 Kenya 2007	F 057 Kenya 2007	F 057 Kenya 2007	F 057 Kenya 2007	Tambul Egypt 1994	Tambul Egypt 1994	20368 South Africa 1981	825/79 Zimbabwe 1979	CAR R 1662 CA. Rep. 1985	AR 21229 Saudi Arabia 2000	Sandfly Fever	neg.	neg.			
lineage	C	C	C	C	C	A	A	F	C	G	C	−	−	−			
[gen cop/ml]	4,8E+06	1,7E+06	3,4E+05	1,9E+05	8,8E+04	3,2E+05	4,3E+04	1,7E+06	2,9E+06	7,2E+05	3,3E+05	−	−	−			
lab n°	#2	#9	#12	#4	#14	#5	#13	#1	#6	#15	#3	#11	#7	#8	score	classification	method
**#26**	+ *	+ *	+ *	+ *	+ *	+ *	+ *	+	+	+ *	+ *	−	−	−	14	Optimal	qRT (23)
**#27b**	+	+	+	+	+	+	+	+	+	+	+	−	−	−	14	Optimal	RPA (38)
**#28a**	**+**	**+**	**+**	**+**	**+**	**+**	**+**	**+**	**+**	**+**	**+**	−	−	−	14	Optimal	qRT (22) LC
**#28b**	**+**	**+**	**+**	**+**	**+**	**+**	**+**	**+**	**+**	**+**	**+**	−	−	−	14	Optimal	qRT (22) QR
**#30**	**+**	**+**	**+**	**+**	**+**	**+**	**+**	**+**	**+**	**+**	**+**	−	−	−	14	Optimal	qRT (22) LC
**#7b**	**+**	**+**	**+**	**+**	**+**	**+**	**+**	**+**	**+**	**+**	**+**	−	−	−	13	Acceptable	qRT (22) LC (co)
**#14b**	+	+	+	+	+	+	FN	+	+	+	+	−	−	−	13	Acceptable	qRT (34) SC
**#14c**	+	+	+	+	+	+	FN	+	+	+	+	−	−	−	13	Acceptable	nRT (20)
**#17**	**+**	**+**	**+**	**+**	**+**	**+**	FN	**+**	**+**	**+**	**+**	−	−	−	13	Acceptable	qRT (23)
**#18**	**+**	**+**	**+**	**+**	**+**	**+**	FN	**+**	**+**	**+**	**+**	−	−	−	13	Acceptable	qRT (36)
**#21b**	**+**	FN	**+**	**+**	**+**	**+**	**+**	**+**	**+**	**+**	**+**	−	−	−	13	Acceptable	qRT (35)
**#25**	**+**	**+**	**+**	**+**	**+**	**+**	FN	**+**	**+**	**+**	**+**	−	−	−	13	Acceptable	qRT (22)
**#27a**	**+**	**+**	**+**	**+**	**+**	**+**	FN	**+**	**+**	**+**	**+**	−	−	−	13	Acceptable	qRT (34)
**#23**	**+** *	**+** *	**+**	**+**	**+**	**+**	**+**	**+**	**+** *	**+**	**+** *	−	−	FP	13	To improve	qRT (22)
**#22**	**+**	**+**	**+**	**+**	**+**	**+**	**+**	**+**	**+**	**+**	**+**	FP	FP	−	12	To improve	qRT(22)+nRT(20)
**#5a**	+	+	+	+	FN	+	FN	+	+	+	FN	−	−	−	11	To improve	RT-LAMP (24)
**#15**	+ *	+ *	FN	+ *	+ *	FN	FN	+ *	+ *	+ *	+ *	−	−	−	11	To improve	qRT(21)+nRT(20)
**#9**	+	+	PBV+	FN	+	FN	PBV+	FN	+	FN	+	TOSV+	−	FP	9	To improve	nRT (37)
**#29**	+	FN	FN	+	+	FN	FN	FN	+	FN	FN	−	−	−	7	To improve	nRT (20)
**% correct**	**100**	**95**	**95**	**97**	**97**	**92**	**79**	**95**	**100**	**95**	**95**	**97**	**97**	**95**			

+: positive.

−: negative.

bold : quantified result.

* : correct strain.

TOSV: Toscana virus.

PBV: Phlebovirus.

RT: reverse transcription.

qRT: real-time RT-PCR.

nRT: nested RT-PCR.

(co): commercial assay.

(ih): in house assay.

FN: false negative.

FP: false positive.

(22): Sall et al., 2002.

(23): Garcia et al. 2001.

(24): Drosten et al., 2002.

(25): Bird et al., 2007.

(26): Le Roux et al. 2009.

(27): Euler et al. 2012.

(31): Weidmann et al. 2008.

(32): Busquets et al. 2010.

(33): Mweango et al. 2012.

(34): Sanchez-Seco et al. 2003.

SC: SmartCycler from Cepheid.

LC: LightCycler from Roche Applied Science.

QR: qRT-PCR System from Qiagen Rotagen.

RT-LAMP: RT-loop-mediated isothermal amplification assay.

RPA: recombinase polymerase amplification assay.

## Discussion

RVF reference laboratories responded keenly to this EQA study (91% response rate), including laboratories situated in RVFV endemic countries such as South Africa and Saudi Arabia. Nonetheless, there is still a need to encourage more laboratories situated in RVF-endemic areas to participate in quality assurance programs. In fact, the increasing amplitude of this disease in Africa necessitates the rapid recognition of RVF outbreaks and implementation of effective control measures in order to prevent uncontrolled and wider spread of the virus.

Most of the laboratories (93%, 28 out of 30) reported the use of qRT-PCR techniques allowing a rapid detection as well as quantification of the virus genome. This confirms that the use of qRT-PCR has remarkably expanded although it requires expensive equipment. All datasets obtained by qRT-PCR only were scored with 13 or 14 points indicating an evenly high performance of all qRT-PCR procedures performed by the different laboratories.

Protocols from Drosten et al, 2002 [24], Bird et al. 2007 [25], Weidmann et al 2008 [31] as well as all in-house qRT-PCR protocols (dataset #6b, #10 and #24) have demonstrated the capacity of providing optimal performances indicating a good specificity and sensitivity for these techniques. The sets of results obtained by applying the qRT-PCR protocols of Mweango et al. 2012 [35], Garcia et al. 2001 [23] and Busquets et al. 2010 [32] did not achieve optimal performances (scores 13, 11 and 13 respectively) but these techniques are not sufficiently represented to conclude on their overall performances.

Information on the viral load of RVFV in human samples can be very useful to monitor the progress of clinical manifestations and to study the pathogenesis of RVFV. Interestingly, not all laboratories employing qRT-PCR techniques have reported quantified results and most of them (64%) reported the results as cycle thereshold (Ct) values and not the number of genome copies. This indicates that most laboratories do not resort to RVFV standards while performing qRT-PCR although such standards would allow them to quantify viral genome in each sample without performing any additional assay. Accordingly to the results of this EQA as well as previous EQA studies, there is still room for improvement concerning viral load determination [29], [30].

The most widely used technique after qRT-PCR was nested RT-PCR with 5 laboratories which referred to 2 different protocols [22], [34]. Nested RT-PCR performances varied greatly compared to qRT-PCR with scores ranging from 7 to 13 thus never reaching optimal performances. The dataset #14c obtained a score of 13 with the protocol of Sall et al. 2002 [22] because it could not detect the highest dilution of the RVFV-Egypt/1994 strain indicating a slightly low sensitivity just as observed for some of the qRT-PCR methods. Nevertheless other datasets referring to nested RT-PCR (#9 and #22) also reported false positive results indicating a lack of specificity of these procedures with both nested RT-PCR protocols [22], .

It is interesting to notice the appearance of newly developed techniques which are suitable for rapid field diagnostics such as RT-LAMP developed in 2009 [26] and RPA technology developed in 2012 [27]. No general conclusion can be achieved concerning the performances of these two techniques as they both have been performed by only one laboratory. However RPA has shown optimal results for this EQA demonstrating equivalent sensitivity and specificity to the qRT-PCR techniques (dataset #27b).

On the other hand, RT-LAMP results indicated difficulties in detecting RVFV genome in the less concentrated samples of the panel (sample #4, #13 and #14). These results suggest some limitations in test sensitivity. However, very high test sensitivity is not essential for field diagnostics in an outbreak situation where most diagnosed patients are in the acute phase of the disease and are expected to present a high viremia.

Three laboratories have provided different sets of results which referred to the same technique and protocol but using different instruments (datasets #5b/c, #14a/b and #28 a/b). These datasets provided all optimal results by using two different instruments except for dataset #14 which reported a slightly lower sensitivity using the SmartCycler System from Cepheid (#14b, 13 points) compared to the 7500 Real-Time PCR System from Applied Biosystems (#14a, 14 points). However, this difference cannot be attributed with certainty to the use of a different instrument as result variability can also arise from a lack of repeatability of the procedure.

Only a few participants provided complete or partial information regarding strain typing (13%, 4 out of 30). However, correct results without strain or genetic lineage specification are satisfactory in the context of laboratory diagnosis. Nonetheless, RVFV strain typing is relevant for surveillance activities in order to monitor which strains are circulating in RVFV-endemic areas and what type of clinical manifestations are associated with these strains.

Comparing the results of this EQA panel to previous EQA studies [29], [30], [36], we observe a higher concordance in terms of performance within laboratories using the same type of diagnostic method. In fact, all qRT-PCR techniques demonstrated an overall good performance with scores ranging from 13 to 14. On the other hand, nested RT-PCR methods have shown a common need for improvement in terms of test sensitivity and/or specificity.

Nevertheless, variations in performance between laboratories using the same method were noted. The reason for such variations is difficult to establish but can be minimized by standardizing procedures, including controls and testing conditions.

In order to ensure optimal performances for RVFV molecular diagnosis in expert laboratories, we recommend conducting EQA studies on a regular basis. Future EQA studies should include a wide range of RVFV isolates with limiting concentrations to assess as precisely as possible the diagnostic performances of various molecular protocols in different reference laboratories.
